# Clonal Spread of Extended-Spectrum Cephalosporin-Resistant *Enterobacteriaceae* Between Companion Animals and Humans in South Korea

**DOI:** 10.3389/fmicb.2019.01371

**Published:** 2019-06-18

**Authors:** Jun Sung Hong, Wonkeun Song, Hee-Myung Park, Jae-Young Oh, Jong-Chan Chae, Saeam Shin, Seok Hoon Jeong

**Affiliations:** ^1^Department of Laboratory Medicine, Research Institute of Bacterial Resistance, Yonsei University College of Medicine, Seoul, South Korea; ^2^Department of Laboratory Medicine, Hallym University College of Medicine, Chuncheon, South Korea; ^3^Department of Veterinary Internal Medicine, Konkuk University College of Veterinary Medicine, Seoul, South Korea; ^4^Division of Biotechnology, Chonbuk National University, Iksan, South Korea

**Keywords:** *Enterobacteriaceae*, ESBL, AmpC, companion animal, human, environment

## Abstract

Extended-spectrum cephalosporin (ESC)-resistant *Enterobacteriaceae* is an increasingly important problem in both human and veterinary medicine. The aims of this study were to describe a comparative molecular characterization of *Enterobacteriaceae* carrying ESC resistance genes, encoding extended-spectrum β-lactamase (ESBL) and AmpC, isolated from human stool samples, rectal swabs from companion animals, and swabs from the environment of veterinarian hospitals in South Korea, and to examine their possible dissemination and transmission. The ESC resistance genes were identified by PCR and sequencing. Isolates with the predominant ESC resistance genes were assessed for their genetic relatedness by pulsed-field gel electrophoresis (PFGE) and multi-locus sequence typing. A total of 195 *Escherichia coli* and 41 *Klebsiella pneumoniae* isolates that exhibited ESC resistance were recovered on CHROMagar ESBL from human, companion animal, and the veterinary hospital environmental samples. In companion animals, most of the ESC resistance genes were *bla*_CMY–2–like_ (26.4%), followed by *bla*_CTX –M–55_ (17.2%) and *bla*_CTX–M–14_ (16.1%), whereas *bla*_CTX–M–15_ (28.6%) was predominant in human samples. The epidemiological relatedness of isolates carrying ESC resistance genes, including 124 *E. coli* and 23 *K. pneumoniae* isolates carrying CMY-2-like, DHA-1-like, or/and CTX-M-type, were analyzed by PFGE. The pulsotypes of five *E. coli* isolates (three from dogs and two from humans) carrying *bla*_CMY–2–like_, which were attributed to sequence type 405, from different veterinary clinics showed >85% similarity. Our results indicate direct transmission and dissemination of ESC-resistant *Enterobacteriaceae* between humans and companion animals.

## Introduction

The concept of “One Health,” which is the integration of human, animal, environmental, and ecosystem health, has recently emerged (Takashima and Day, 2014). One issue that should be addressed through a One Health approach is antimicrobial resistance (AMR). Therefore, we need to take steps to address the dissemination of AMR through the adoption of a One Health approach, promoting the integration of human and animal health, food safety, and environmental surveillance (Roca et al., 2015; Sikkema and Koopmans, 2016).

*Enterobacteriaceae* are among the most commonly reported causes of bacterial infections in humans and animals (Schmiedel et al., 2014). Notably, *Enterobacteriaceae* carrying extended-spectrum β-lactamases (ESBLs) and AmpC β-lactamases (AmpCs) are broadly distributed among extended-spectrum cephalosporin (ESC)-resistant bacteria (Kameyama et al., 2013). In both humans and animals, CTX-M-type enzymes are the most common ESBLs, whereas CMY-, and DHA-type enzymes are the most prevalent plasmid-mediated AmpCs (Liebana et al., 2004; Jacoby, 2009; Matsumura et al., 2012). These enzymes are able to inactivate ESCs and are normally encoded on mobile genetic elements, thus they can be transmitted to the same or different bacteria in humans, animals, foods, or the environment through either directly (transmission of AMR bacteria), or indirectly (transfer of AMR genes) (Haenni et al., 2014; Hong et al., 2016). Companion animals could play a role as a reservoir of AMR bacteria, as they are in close association with humans, living in their homes, and near their food. In addition, the widespread use and misuse of antibiotics in both human and veterinary medicine is increasing the spread of AMR bacteria (Dorado-García et al., 2018; Melo et al., 2018; Pulss et al., 2018). However, the impact of companion animals on human health in terms of attributing to exchange and share AMR determinant is not yet clear. Therefore, systematic control and prevention, through implementation of a national AMR surveillance program, are greatly needed and should be applied in the fields of human, and veterinary clinical medicine.

In this study, we represented a molecular characterization of ESC-resistant *Enterobacteriaceae* isolates collected from companion animals, humans, and veterinary hospital environments as part of a national surveillance program at 36 veterinary hospitals in South Korea and examined their epidemiological relatedness.

## Materials and Methods

### Bacterial Profiles

We collected *Escherichia coli* and *Klebsiella pneumoniae* isolates from the rectal swabs of companion animals, including dogs (*n* = 315) and cats (*n* = 74); stool samples of humans, including pet owners (*n* = 48) and medical staff (*n* = 33); and 352 swabs of veterinary hospital environmental surfaced, including examination tables, cages, water bowls, scales, microscopes, keyboards, and switches, at 36 veterinarian hospitals of various regions in South Korea during July to November, 2017. All samples were cultured on CHROMagar ESBL (CHROMagar, Paris, France) for use in the selection of *E. coli* (dark pink to reddish colony) and *Klebsiella*/*Enterobacter*/*Citrobacter* species (metallic blue colony). After pure sub-culture for a single colony on each blood agar plate (SPL Life Sciences, Gyeonggi-do, South Korea) per a given sample, *E. coli* and *K. pneumoniae* isolates were selected and identified by matrix-assisted laser desorption ionization-time of flight mass spectrometry (MALDI-TOF MS) with a Vitek-MS (bioMérieux, Marcy-l’Etoile, France).

### Antimicrobial Susceptibility Testing

The isolates were tested for antimicrobial susceptibility to the following antimicrobial agents: ampicillin, piperacillin, ampicillin-sulbactam, cefazolin, cefoxitin, cefotaxime, ceftazidime, cefepime, aztreonam, ertapenem, imipenem, meropenem, amikacin, gentamicin, ciprofloxacin, and trimethoprim-sulfamethoxazole, by the agar disk diffusion method on Mueller-Hinton agar (Difco Laboratories, Detroit, MI, United States) according to the guidelines of the Clinical and Laboratory Standards Institute (CLSI, 2018b). The minimal inhibitory concentration of colistin was determined by broth microdilution using the criteria of the European Committee for Antimicrobial Susceptibility Testing (EUCAST, 2018).

### Detection of Antimicrobial Resistance Genes

Template DNA was prepared by the boiling method. PCR and DNA sequencing of various AMR genes, including *bla*_CTX–M–1 group_, *bla*_CTX–M–2 group_, *bla*_CTX–M–9 group_, *bla*_CTX–M–25 group_, *bla*_TEM_, *bla*_SHV_, *bla*_DHA_, *bla*_CMY–1_, *bla*_CMY–2_, *bla*_ACC_, *bla*_ACT_, *bla*_FOX_, *bla*_IMP_, *bla*_VIM_, *bla*_NDM_, *bla*_KPC_, *bla*_GES_, and *bla*_OXA–48–like_, were performed as described previously (Lee et al., 2018a). The sequences were compared to published DNA sequences using BLAST^1^.

### ESBL/AmpC Production Phenotypic Testing

ESC-resistant isolates with ESBL/AmpC PCR-negative were performed phenotypic disk diffusion test to confirm for ESBL/AmpC production (Song et al., 2007a, b).

### Nucleotide Sequence-Based Bacterial Typing

The epidemiological relationships among the CTX-M-type or/and CMY-2-like-producing isolates, which being collected from humans, companion animals, and the environment, were analyzed by pulsed-field gel electrophoresis (PFGE) using *Xba*I restriction enzyme. Then after, multilocus sequence typing (MLST) was performed for *E. coli* and *K. pneumoniae* strains with representative PFGE profiles as described previously (Nemoy et al., 2005; Jeong et al., 2016). *E. coli* ATCC 25922 and *K. pneumoniae* ATCC 13883 were included as quality controls.

### Statistical Analysis

Statistical analysis was performed using SPSS Statistics version 24.0.0 (IBM Corp., Armonk, NY, United States). For comparison of the ESC-resistance rates between groups, we used the chi-square test. All *p* values were two-sided, and values less than 0.05 were considered statistically significant.

## Results

### ESC Resistance and Antimicrobial Susceptibility

Among 389 samples of companion animals (315 dogs and 74 cats), 81 of humans (48 owners and 33 staffs), and 352 of veterinary hospital environmental surface, a total of 236 non-duplicated ESC-resistant isolates, including 195 *E. coli*, and 41 *K. pneumoniae* isolates were recovered. There were some cases that both ESC-resistant *E. coli* and *K. pneumoniae* isolates were simultaneously selected from 17 dogs, one cat, and one the environmental sample (Supplementary Table S1). Of the 195 ESC-resistant *E. coli* isolates, 174 (44.7%) were recovered from companion animals (389 total samples), 14 (17.3%) were recovered from human stool samples (81 total samples), and 7 (2%) were recovered from the environment (352 total samples). Among the *E. coli* isolates from companion animals, the ESC-resistance rate for canines (49.2%) was significantly higher than the rate for feline isolates (25.7%) (*p* value = <0.001). Of the 41 ESC-resistant *K. pneumoniae* isolates, 29 (7.5%) were recovered from companion animals, and 12 (3.4%) were recovered from the environment. Among the *K. pneumoniae* isolates from companion animals, the ESC-resistance rate for canines (8.3%) was not statistically different from the rate of felines (4.1%) (*p* value = 0.216). None were recovered from human-derived samples (Table 1). The AMR profiles of the ESC-resistant *E. coli* and *K. pneumoniae* isolates are listed in Supplementary Tables S2, S3, respectively.

**TABLE 1:** Genotypes of β-lactamases in extended-spectrum cephalosporin-resistant *E. coli* and *K. pneumoniae* isolates from companion animals, humans, and the hospital environment.

**Organism**	**ESC resistance gene^a^**			**No. (%) isolates**				
	**ESBL^b^**	**AmpC**	**Carbapenemase**	**Companion animal**		**Human (*n* = 81)**	**Environment (*n* = 352)**	**Total (*n* = 822)**
				**Dog (*n* = 315)**	**Cat (*n* = 74)**			
*E. coli*	CTX-M-1			3	1	0	0	4
	CTX-M-3			4	0	0	1	5
	CTX-M-15			11	1	4	0	16
	CTX-M-15	CMY-2-like	NDM-5	4	0	0	0	4
	CTX-M-55			26	1	0	0	27
	CTX-M-55 + CTX-M-14			0	1	0	0	1
	CTX-M-55	CMY-2-like		2	0	0	0	2
	CTX-M-14			25	2	1	1	29
	CTX-M-14	CMY-2-like		1	0	0	0	1
	CTX-M-14	DHA-1-like		1	0	0	0	1
	CTX-M-24			3	2	0	0	5
	CTX-M-27			5	1	2	0	8
	CTX-M-65			6	1	0	0	7
	SHV-190			1	0	0	0	1
	CMY-2-like		40	6	2	3	51	
		CMY-2-like + DHA-1-like		0	2	0	0	2
	CMY-4		1	0	0	0	1	
		DHA-1-like		3	0	2	0	5
		DHA-9		1	0	0	0	1
	Unidentified	Unidentified	Unidentified	18	1	3	2	24
	Total			155 (49.2)	19 (25.7)	14 (17.3)	7 (2.0)	195 (23.7)
*K. pneumoniae*	CTX-M-15			10	1	0	1	12
	CTX-M-15	DHA-1-like		6	0	0	6	12
	CTX-M-15 + CTX-M-14	DHA-1-like		1	0	0	0	1
	CTX-M-55	DHA-1-like		0	0	0	1	1
	CTX-M-55	DHA-9		0	0	0	1	1
	CTX-M-14			2	0	0	1	3
	SHV-12	DHA-1-like		2	0	0	2	4
	SHV-26	DHA-1-like		2	0	0	0	2
	SHV-28			1	0	0	0	1
		CMY-2-like		1	1	0	0	2
		DHA-1-like		0	1	0	0	1
		CMY-2-like + DHA-1-like		1	0	0	0	1
	Total			26 (8.3)	3 (4.1)	0 (0)	12 (3.4)	41 (5.0)

*^a^ESC, extended-spectrum cephalosporin.^b^ESBL, extended-spectrum β-lactamase.*

### Genotypes of ESC Resistance

We performed genotype characterization of ESC resistance for all ESC-non-susceptible 195 *E. coli* and 41 *K. pneumoniae* isolates, regardless of isolation of both *E. coli* and *K. pneumoniae* isolates in same sample. Among the ESC-non-susceptible 195 *E. coli*, 171 (87.7%) isolates harbored known ESC resistance genes (Table 1), including nine different ESBL types and four AmpC types. A few isolates harbored both ESBL and AmpC (seven isolates carried CTX-M-type and CMY-2-like genes, and one isolate carried CTX-M-type and DHA-1-like genes). Of the 174 ESC-resistant *E. coli* isolates categorized from companion animals, *bla*_CMY–2–like_ gene (*n* = 46, 26.4%) was most common ESC resistance determinant, followed by CTX-M-55 (*n* = 30, 17.2%), CTX-M-14 (*n* = 28, 16.1%), and CTX-M-15 (*n* = 20, 11.5%), which were the dominant ESBL genes. Interestingly, for the ESC-resistant *E. coli* isolates in companion animals, NDM-5 was detected in four isolates along with *bla*_CTX–M–15_ and *bla*_CMY–2–like_. Of the 14 ESC-resistant *E. coli* isolates from humans, CTX-M-15 was predominant, which accounted for 28.6% of the total (*n* = 4). One isolate was positive for SHV-190, and remaining 24 isolates were negative for both known ESBLs and AmpCs by the primers used in this study.

Among the ESC-non-susceptible 41 *K. pneumoniae* isolates, six different ESBL types and three AmpC types were detected in all 41 (100%) isolates (Table 1). In samples from companion animals and the environment, CTX-M-15 (*n* = 25) and DHA-1-like (*n* = 21) were dominant genotypes, followed by SHV-types (*n* = 5), and CTX-M-14 (*n* = 5). The presence of both ESBL- and AmpC-type genes was detected in 21 *K. pneumoniae* isolates (51.2%), and in 8 *E. coli* isolates (4.1%). No ESC-resistant *K. pneumoniae* isolates were recovered from human stool samples (Table 1).

### Macro-Restriction of ESC-Resistant *Enterobacteriaceae*

**FIGURE 1:**
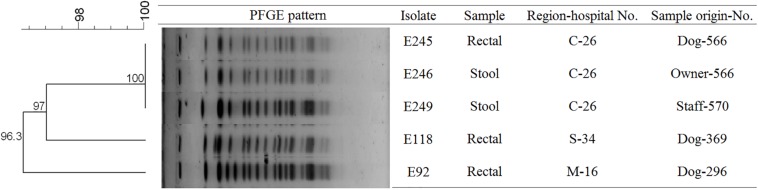
Pulsed-field gel electrophoresis (PFGE) dendrogram of the five CMY-2-like producing *E. coli* ST405 (This image was modified for illustrative purposes). Owner-566 is a veterinarian in the hospital C-26. Staff-570 is a nurse in the hospital C-26. M, metropolitan region; C, central region; S, southern region.

**FIGURE 2:**
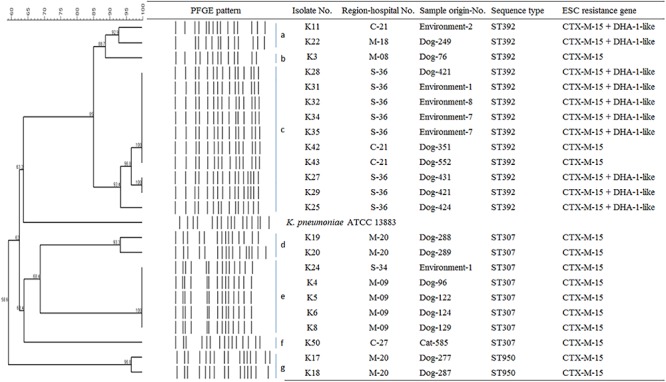
Epidemiological profiles of 23 *K. pneumoniae* isolates carrying ESC resistance gene (CTX-M-15 and DHA-1-like) determined by PFGE analysis using *Xba*I restriction. Environment-1, examining table; Environment-2, cage; Environment-4, scale; Environment-7, keyboard; Environment-8, switch; M, metropolitan region; C, central region; S, southern region.

To determine the epidemiological relatedness among ESC-resistant isolates from companion animals, humans, and various hospital areas in veterinarian clinics, 124 *E. coli* and 23 *K. pneumoniae* isolates carrying the predominant ESC resistance genes (*E. coli* carrying CTX-M-15, CTX-M-55, CTX-M-14, or/and CMY-2-like genes, and *K. pneumoniae* carrying CTX-M-15 or/and DHA-1-like genes) were subjected to PFGE analysis. Among the 124 *E. coli* isolates, similar PFGE patterns were observed only between isolates from companion animals and humans (Supplementary Figure S1). Five CMY-2-like harboring *E. coli* isolates without other resistance genes belonged to one pulsotype, with >85% similarity, which was attributed to sequence type 405 (ST405) by MLST analysis. Three of the five isolates were derived from samples obtained at the same hospital, two were from humans (E246 was from a veterinarian and E249 was from a nurse) and the third was from a rectal swab from a dog (E245) admitted to the above hospital. The two remaining isolates (E92 and E118) were collected from two dogs at different veterinary clinics (Figure 1). For the 23 *K. pneumoniae* isolates that mostly produced CTX-M-15 or/and DHA-1-like enzymes in companion animals and the environment, 7 pulsotypes were identified which corresponded to ST307 (d, e, and f PFGE-types), ST392 (a, b, and c PFGE-types), and ST950 (g PFGE-type) (Figure 2).

## Discussion

Antimicrobial resistance surveillance systems of clinical isolates in South Korea have been well-documented in both the Korea antimicrobial resistance monitoring system (KARMS) and Korea global antimicrobial surveillance system (Kim et al., 2017; Lee et al., 2018b). However, this is the first report on the prevalence and molecular characterization of ESC-resistant *Enterobacteriaceae* isolates collected from companion animals, humans, and the veterinarian hospital environment for national surveillance in South Korea. Previously, the KARMS study for human clinical isolates reported that the rate of AMR to cefotaxime was 35.0% in *E. coli* and 41.0% in *K. pneumoniae* (Kim et al., 2017). Among the *E. coli* isolates collected from rectal swabs of dogs and cats obtained in this study, the rates of ESBL- or/and AmpC-producing *E. coli* isolates were 43.5% (137/315) for dogs and 24.3% (18/74) for cats, respectively. In previous study showed that the rate of ESBL or/and AmpC-producing *E. coli* isolates in dogs had a 38.1% (So et al., 2012), which represented currently increasing incidence in South Korea.

CTX-M type ESBL genes in the fields of human medicine, and CMY type AmpC genes in both human and veterinary medicine, are dominant in *E. coli* worldwide including South Korea (Kameyama et al., 2013; Lee et al., 2018a). Especially, CTX-M-15- and CMY-2-producing *E. coli* isolates are the most frequently detected genotypes associated with ESC resistance in companion animals and humans, respectively (Matsumura et al., 2012; Woerther et al., 2013; Haenni et al., 2014; Dorado-García et al., 2018). Because companion animals are in close contact with humans, the genotypes of companion animals in this study were initially expected to be similar to the genotypes of humans disseminated in South Korea. The total number of CMY-2-like-producing *E. coli* isolates was greater than the number of CTX-M-type (CTX-M-55-, CTX-M-14, and CTX-M-15)-producing isolates from the rectal swabs of companion animals, which was consistent with the numbers described in previous reports of AmpC in animals, suggesting that it is an important mechanism of resistance to ESC (So et al., 2012; Dorado-García et al., 2018). Instead, CTX-M-15 was more detected than other ESC-resistant determinants in humans, but CTX-M-55 and CTX-M-14 were more prevalent than CTX-M-15 in companion animals in this study. The CTX-M-55 and CTX-M-14 were previously detected in food-producing animals and turkey meat, respectively (Kiratisin et al., 2008; Randall et al., 2011; Matsumura et al., 2012; Liao et al., 2015). These findings indicate that the ESC resistance gene variants are not limited to certain hosts, emphasizing the need for coordinated control in the concept of One Health. In addition, in previous studies, CTX-M-14 were the most common ESC resistance genotypes, whereas CTX-M-55 was rarely detected in companion animals in South Korea (So et al., 2012; Tamang et al., 2012). Therefore, the observed increase in CTX-M-55 in companion animals in South Korea may also affect AMR transmission between humans and companion animals.

All 41 ESC-resistant *K. pneumoniae* isolates harbored ESC-resistance genes. In contrast, of the 195 ESC-resistant *E. coli* isolates, 24 (12.3%) isolates did not identify for ESBL, and/or AmpC type in this study. These isolates were susceptible to cefoxitin and were positive for ESBL production (disk diffusion test), suggesting the presence of an ESBL variant not detected by the primers used in this study. We performed PFGE to understand the relatedness between ESC-resistant *E. coli* and *K. pneumoniae* isolates from humans, companion animals, and the environment in veterinary hospitals during 5 months. Most of the ESC-resistant isolates had no evidence of clonal spread between humans and companion animals. Instead, the five CMY-2-producing *E. coli* ST405 isolates from two humans and three dogs showed high identity, which suggested the possibility of direct transmission between humans, and companion animals. The spread of *E. coli* ST405 carrying CTX-M-15 has been frequently described in humans as an epidemic lineage, along with *E. coli* ST131 (Coque et al., 2008). However, we detected *E. coli* ST405 carrying CMY-2-like genes in this study.

In this study, CTX-M-15 (61.0%, *n* = 25/41), either alone or in combination with DHA-1-like, was mostly detected in ESC-resistant *K. pneumoniae* isolates from companion animals and the environmental samples. Recently, CTX-M-15- and DHA-1-coproducing *K. pneumoniae* ST11 have emerged in human patients and are being disseminated (Cha et al., 2018; Lee et al., 2018a), while there was no report that described in *K. pneumoniae* isolate carrying CTX-M-15 from dogs and cats in South Korea. CTX-M-15 was essentially associated with ST11, ST15, ST307, and ST392 clones in *K. pneumoniae*, which have been frequently detected in other part of the world (Ewers et al., 2014; Wyres et al., 2019). ST307 is also frequent among carbapenemase producers in South Korea, suggesting a wider dissemination in different setting in our country (Yoon et al., 2018a, b). There was no transmission between humans and companion animals, however, clonal spread was observed among companion animals and between companion animals and the environment in this study. Based on the observation of environmental colonization of AMR *K. pneumoniae* in veterinary hospitals, infection control for the environment should be carefully considered in the veterinary field.

Our results have limitations: (i) the number of stool specimens from humans was less than that of companion animals and the isolation of ESBL/AmpC producing *K. pneumoniae* was unusually more frequent than *E. coli* with ESBL/AmpC in the environmental samples. Unfortunately, we do not know the reason why for this, (ii) plasmid distribution was not included in this study, which is well known to contribute to ESBL distribution in *Enterbacteriaceae*. Nevertheless, our findings illustrate the importance of infection control strategies for usage of antibiotics and demonstrate the need for further cooperation among the fields of human and veterinary medicine and environmental science in the One Health perspective.

## Data Availability

Publicly available datasets were analyzed in this study. This data can be found here: https://blast.ncbi.nlm.nih.gov/Blast.cgi.

## Ethics Statement

This study was carried out in accordance with the recommendation of ethical guidelines of KonKuk University College of Veterinary Medicine, South Korea. Individual written informed consent for the use of samples was obtained from all the animal owners and veterinarian.

## Author Contributions

WS was responsible for the study design, data analysis, and proofreading of the manuscript. JH performed examination of molecular work (resistance gene, PFGE, and MLST), analyzed the experimental data, and wrote the manuscript. H-MP, J-CC, and SJ designed the sample collection and experiments. J-YO designed and performed the experiments. SS conducted the statistical analysis and wrote the manuscript.

## Conflict of Interest Statement

The authors declare that the research was conducted in the absence of any commercial or financial relationships that could be construed as a potential conflict of interest.

## Footnotes

1 https://blast.ncbi.nlm.nih.gov/Blast.cgi

## Supplementary Material

FIGURE S1 Epidemiological profiles of 124 ESBL/AmpC (CTX-M-15, CTX-M-55, CTX-M-14, or/and CMY-2-like) producing *E. coli* isolates from humans, companion animals (green box), and the environment (blue box) determined by PFGE analysis using XbaI restriction. Red box represented five CMY-2-like producing *E. coli* isolates related closely. *E. coli* ATCC strain used in this study was *E. coli* ATCC 25922.

TABLE S1 Samples that grew both *E. coli* and *K. pneumoniae* isolates from dog, cat, and the environment.

TABLE S2 Antimicrobial susceptibilities of extended-spectrum cephalosporin-resistant *E. coli* isolates from humans, companion animals, and the hospital environment.

TABLE S3 Antimicrobial susceptibilities of extended-spectrum cephalosporin-resistant *K. pneumoniae* isolates from companion animals and the hospital environment.

## References

[B1] Cha M. K. Kang C. I. Kim S. H. Chung D. R. Peck K. R. Lee N. Y. (2018). High Prevalence of CTX-M-15-Type Extended-Spectrum β-Lactamase Among AmpC β-Lactamase-Producing *Klebsiella pneumoniae* Isolates Causing Bacteremia in Korea. *Microb. Drug. Resist.* 24 1002–1005. 10.1089/mdr.2017.0362 29584568

[B2] CLSI. (2018b). *Performance Standards for Antimicrobial Susceptibility Testing; Twenty-eighth Informational Supplement.* Wayne, PA: Clinical and Laboratory Standards Institute.

[B3] Coque T. M. Novais A. Carattoli A. Poirel L. Pitout J. Peixe L. (2008). Dissemination of clonally related *Escherichia coli* strains expressing extended-spectrum beta-lactamase CTX-M-15. *Emerg. Infect. Dis.* 14 195–200. 10.3201/eid1402.070350 18258110 PMC2600198

[B4] Dorado-García A. Smid J. H. van Pelt W. Bonten M. J. M. Fluit A. C. van den Bunt G. (2018). Molecular relatedness of ESBL/AmpC-producing *Escherichia coli* from humans, animals, food and the environment: a pooled analysis. *J. Antimicrob. Chemother.* 73 339–347. 10.1093/jac/dkx397 29165596

[B5] EUCAST (2018). *The European Committee on Antimicrobial Susceptibility Testing. Breakpoint Tables for Interpretation of MICs and Zone Diameters, Version 9.0.* Available at: http://www.eucast.org/clinical_breakpoints/ (accessed January 1, 2019).

[B6] Ewers C. Stamm I. Pfeifer Y. Wieler L. H. Kopp P. A. Schønning K. (2014). Clonal spread of highly successful ST15-CTX-M-15 *Klebsiella pneumoniae* in companion animals and horses. *J. Antimicrob. Chemother.* 69 2676–2680. 10.1093/jac/dku217 24974381

[B7] Haenni M. Châtre P. Métayer V. Bour M. Signol E. Madec J. Y. (2014). Comparative prevalence and characterization of ESBL-producing *Enterobacteriaceae* in dominant versus subdominant enteric flora in veal calves at slaughterhouse, France. *Vet. Microbiol.* 171 321–327. 10.1016/j.vetmic.2014.02.023 24629776

[B8] Hong J. S. Yoon E. J. Lee H. Jeong S. H. Lee K. (2016). Clonal Dissemination of *Pseudomonas aeruginosa* Sequence Type 235 Isolates Carrying *bla*IMP-6 and Emergence of *bla*GES-24 and *bla*IMP-10 on Novel Genomic Islands PAGI-15 and -16 in South Korea. *Antimicrob. Agents. Chemother.* 60 7216–7223. 27671068 10.1128/AAC.01601-16 PMC5119001

[B9] Jacoby G. A. (2009). AmpC beta-lactamases. *Clin. Microbiol. Rev* 22 161–182. 10.1128/CMR.00036-38 19136439 PMC2620637

[B10] Jeong S. H. Kim H. S. Kim J. S. Shin D. H. Kim H. S. Park M. J. (2016). Prevalence and Molecular Characteristics of Carbapenemase-Producing *Enterobacteriaceae* From Five Hospitals in Korea. *Ann. Lab. Med.* 36 529–535. 10.3343/alm.2016.36.6.529 27578505 PMC5011105

[B11] Kameyama M. Chuma T. Yabata J. Tominaga K. Iwata H. Okamoto K. (2013). Prevalence and epidemiological relationship of CMY-2 AmpC β-lactamase and CTX-M extended-spectrum β-lactamase-producing *Escherichia coli* isolates from broiler farms in Japan. *J. Vet. Med. Sci*. 75 1009–1015. 10.1292/jvms.12-0453 23503164

[B12] Kim D. Ahn J. Y. Lee C. H. Jang S. J. Lee H. Yong D. (2017). Increasing Resistance to Extended-Spectrum Cephalosporins, Fluoroquinolone, and Carbapenem in Gram-Negative Bacilli and the Emergence of Carbapenem Non-Susceptibility in *Klebsiella pneumoniae*: Analysis of Korean Antimicrobial Resistance Monitoring System (KARMS) Data From 2013 to 2015. *Ann. Lab. Med.* 37 231–239. 10.3343/alm.2017.37.3.231 28224769 PMC5339095

[B13] Kiratisin P. Apisarnthanarak A. Laesripa C. Saifon P. (2008). Molecular characterization and epidemiology of extended-spectrum-beta-lactamase-producing *Escherichia coli* and *Klebsiella pneumoniae* isolates causing health care-associated infection in Thailand, where the CTX-M family is endemic. *Antimicrob. Agents. Chemother* 52 2818–2824. 10.1128/AAC.00171-178 18505851 PMC2493136

[B14] Lee H. Yoon E. J. Kim D. Jeong S. H. Won E. J. Shin J. H. (2018a). Antimicrobial resistance of major clinical pathogens in South Korea, May 2016 to April 2017: first one-year report from Kor-GLASS. *Euro Surveill* 23 1800047. 10.2807/1560-7917.ES.2018.23.42.1800047 30352640 PMC6199864

[B15] Lee H. Yoon E. J. Kim D. Jeong S. H. Shin J. H. Shin J. H. (2018b). Establishment of the South Korean national antimicrobial resistance surveillance system, Kor-GLASS, in 2016. *Euro Surveill* 23 1700734. 10.2807/1560-7917.ES.2018.23.42.1700734 30352643 PMC6199867

[B16] Liao X. P. Xia J. Yang L. Li L. Sun J. Liu Y. H. (2015). Characterization of CTX-M-14-producing *Escherichia coli* from food-producing animals. *Front. Microbiol.* 6:1136. 10.3389/fmicb.2015.01136 26528278 PMC4606122

[B17] Liebana E. Batchelor M. Clifton-Hadley F. A. Davies R. H. Hopkins K. L. Threlfall E. J. (2004). First report of *Salmonella* isolates with the DHA-1 AmpC beta-lactamase in the United Kingdom. *Antimicrob. Agents. Chemother.* 48 4492. 10.1128/AAC.48.11.4492.2004 15504894 PMC525448

[B18] Matsumura Y. Yamamoto M. Nagao M. Hotta G. Matsushima A. Ito Y. (2012). Emergence and spread of B2-ST131-O25b, B2-ST131-O16 and D-ST405 clonal groups among extended-spectrum-β-lactamase-producing *Escherichia coli* in Japan. *J. Antimicrob. Chemother.* 67 2612–2620. 10.1093/jac/dks278 22843833

[B19] Melo L. C. Oresco C. Leigue L. Netto H. M. Melville P. A. Benites N. R. (2018). Prevalence and molecular features of ESBL/pAmpC-producing *Enterobacteriaceae* in healthy and diseased companion animals in Brazil. *Vet. Microbiol.* 221 59–66. 10.1016/j.vetmic.2018.05.017 29981709

[B20] Nemoy L. L. Kotetishvili M. Tigno J. Keefer-Norris A. Harris A. D. Perencevich E. N. (2005). Multilocus sequence typing versus pulsed-field gel electrophoresis for characterization of extended-spectrum beta-lactamase-producing *Escherichia coli* isolates. *J. Clin. Microbiol.* 43 1776–1781. 10.1128/jcm.43.4.1776-1781.2005 15814998 PMC1081380

[B21] Pulss S. Stolle I. Stamm I. Leidner U. Heydel C. Semmler T. (2018). Multispecies and Clonal Dissemination of OXA-48 Carbapenemase in *Enterobacteriaceae* From Companion Animals in Germany, 2009-2016. *Front. Microbiol.* 9:1265. 10.3389/fmicb.2018.01265 29963026 PMC6010547

[B22] Randall L. P. Clouting C. Horton R. A. Coldham N. G. Wu G. Clifton-Hadley F. A. (2011). Prevalence of *Escherichia coli* carrying extended-spectrum β-lactamases (CTX-M and TEM-52) from broiler chickens and turkeys in Great Britain between 2006 and 2009. *J. Antimicrob. Chemother.* 66 86–95. 10.1093/jac/dkq396 21098542

[B23] Roca I. Akova M. Baquero F. Carlet J. Cavaleri M. Coenen S. (2015). The global threat of antimicrobial resistance: science for intervention. *New. Microbes. New. Infect.* 6 22–29. 10.1016/j.nmni.2015.02.007 26029375 PMC4446399

[B24] Schmiedel J. Falgenhauer L. Domann E. Bauerfeind R. Prenger-Berninghoff E. Imirzalioglu C. (2014). Multiresistant extended-spectrum β-lactamase-producing *Enterobacteriaceae* from humans, companion animals and horses in central Hesse, Germany. *BMC Microbiol* 14:187. 10.1186/1471-2180-14-187 25014994 PMC4105247

[B25] Sikkema R. Koopmans M. (2016). One Health training and research activities in Western Europe. *Infect. Ecol. Epidemiol.* 6 33703. 10.3402/iee.v6.33703 27906121 PMC5131506

[B26] So J. H. Kim J. Bae I. K. Jeong S. H. Kim S. H. Lim S. K. (2012). Dissemination of multidrug-resistant *Escherichia coli* in Korean veterinary hospitals. *Diagn. Microbiol. Infect. Dis.* 73 195–199. 10.1016/j.diagmicrobio.2012.03.010 22516765

[B27] Song W. Bae I. K. Lee Y. N. Lee C. H. Lee S. H. Jeong S. H. (2007a). Detection of extended-spectrum beta-lactamases by using boronic acid as an AmpC beta-lactamase inhibitor in clinical isolates of *Klebsiella* spp. *and Escherichia coli*. *J. Clin. Microbiol.* 45 1180–1184. 10.1128/jcm.02322-06 17301276 PMC1865824

[B28] Song W. Jeong S. H. Kim J. S. Kim H. S. Shin D. H. Roh K. H. (2007b). Use of boronic acid disk methods to detect the combined expression of plasmid-mediated AmpC beta-lactamases and extended-spectrum beta-lactamases in clinical isolates of *Klebsiella* spp., *Salmonella spp., and Proteus mirabilis*. *Diagn. Microbiol. Infect. Dis.* 57 315–318. 10.1016/j.diagmicrobio.2006.08.023 17174510

[B29] Takashima G. K. Day M. J. (2014). Setting the One Health agenda and the human-companion animal bond. *Int J. Environ. Res. Public. Health.* 11 11110–11120. 10.3390/ijerph111111110 25350006 PMC4245602

[B30] Tamang M. D. Nam H. M. Jang G. C. Kim S. R. Chae M. H. Jung S. C. (2012). Molecular characterization of extended-spectrum-β-lactamase-producing and plasmid-mediated AmpC β-lactamase-producing *Escherichia coli* isolated from stray dogs in South Korea. *Antimicrob. Agents. Chemother* 56 2705–2712. 10.1128/AAC.05598-5511 22354297 PMC3346616

[B31] Woerther P. L. Burdet C. Chachaty E. Andremont A. (2013). Trends in human fecal carriage of extended-spectrum β-lactamases in the community: toward the globalization of CTX-M. *Clin. Microbiol. Rev* 26 744–758. 10.1128/CMR.00023-13 24092853 PMC3811232

[B32] Wyres K. L. Hawkey J. Hetland M. A. K. Fostervold A. Wick R. R. Judd L. M. (2019). Emergence and rapid global dissemination of CTX-M-15-associated *Klebsiella pneumoniae* strain ST307. *J. Antimicrob. Chemother.* 74 577–581. 10.1093/jac/dky492 30517666 PMC6376852

[B33] Yoon E. J. Kim J. O. Kim D. Lee H. Yang J. W. Lee K. J. (2018a). *Klebsiella pneumoniae* Carbapenemase Producers in South Korea between 2013 and 2015. *Front. Microbiol.* 9:56. 10.3389/fmicb.2018.00056 29422888 PMC5788937

[B34] Yoon E. J. Yang J. W. Kim J. O. Lee H. Lee K. J. Jeong S. H. (2018b). Carbapenemase-producing *Enterobacteriaceae* in South Korea: a report from the National Laboratory Surveillance System. *Future. Microbiol* 13 771–783. 10.2217/fmb-2018-2022 29478336

