# Supplementary figures and images for: Clonal Spread of Extended-Spectrum Cephalosporin-Resistant Enterobacteriaceae Between Companion Animals and Humans in South Korea
Source: Front Microbiol. 2019 Jun 18;10:1371. doi: 10.3389/fmicb.2019.01371 (PMC6591270; doi:10.3389/fmicb.2019.01371)

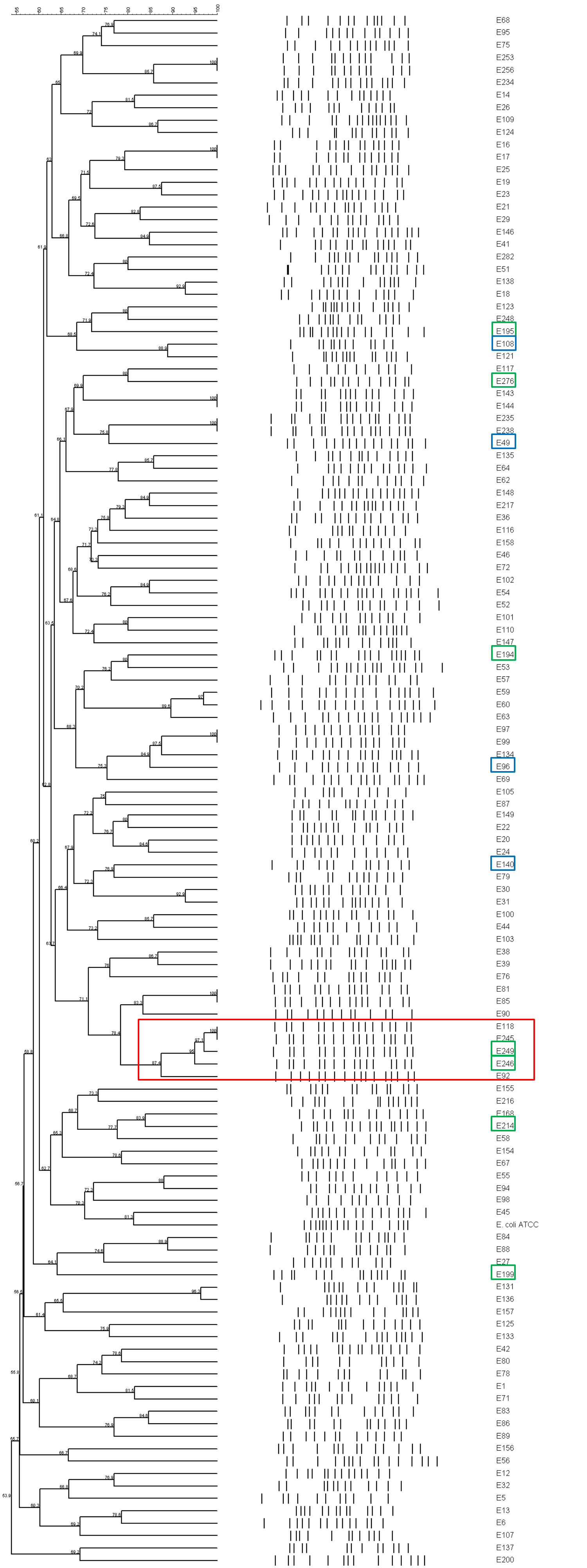

Supplement: FIGURE S1 — Epidemiological profiles of 124 ESBL/AmpC (CTX-M-15, CTX-M-55, CTX-M-14, or/and CMY-2-like) producing E. coli isolates from humans, companion animals (green box), and the environment (blue box) determined by PFGE analysis using XbaI restriction. Red box represented five CMY-2-like producing E. coli isolates related closely. E. coli ATCC strain used in this study was E. coli ATCC 25922. [file Image_1.TIF]
