# Supplementary material for: Clonal Spread of Extended-Spectrum Cephalosporin-Resistant Enterobacteriaceae Between Companion Animals and Humans in South Korea
Source: Front Microbiol. 2019 Jun 18;10:1371. doi: 10.3389/fmicb.2019.01371 (PMC6591270; doi:10.3389/fmicb.2019.01371)
Supplement: Supplementary file 5 [file Data_Sheet_1.PDF]

FIG S1 Epidemiological profiles of 124 ESBL/AmpC (CTX-M-15, CTX-M-55, CTX-M-14, or/and CMY-2-like) producing *E. coli* isolates from humans, companion animals (green box), and the environment (blue box) determined by PFGE analysis using *Xba*I restriction. Red box represented five CMY-2-like producing *E. coli* isolates related closely. *E. coli* ATCC strain used in this study was *E. coli* ATCC 25922.
